# Mucinous adenocarcinoma of the appendix with inguinal node metastases

**DOI:** 10.4322/acr.2021.335

**Published:** 2021-11-12

**Authors:** Ricardo Vaz-Pereira, Rita Marques, Urânia Fernandes, Ana Monteiro, João Pinto-de-Sousa

**Affiliations:** 1 Centro Hospitalar de Trás-Os-Montes e Alto Douro (CHTMAD), Serviço de Cirurgia Geral, Vila Real, Portugal

**Keywords:** Appendix, Adenocarcinoma Mucinous, Lymphatic Metastasis, Groin

## Abstract

Mucinous adenocarcinoma of the appendix is a rare neoplasm with a low propensity for lymph node metastasis. The present case refers to an appendicular mucinous adenocarcinoma with inguinal lymph node metastasis. A 71-year-old woman underwent an appendectomy due to a clinical presentation of acute appendicitis. However, the histological examination of the surgical specimen revealed a mucinous adenocarcinoma of the appendix. After staging, the patient underwent a right hemicolectomy and was proposed for adjuvant chemotherapy. At the 3rd year of follow-up, inguinal lymphadenopathy was diagnosed, which biopsy confirmed inguinal node metastases from primary colorectal cancer, with areas of extracellular mucin. Restaging revealed liver and peritoneal metastasis, and the patient was proposed for palliative chemotherapy. Appendicular neoplasms, due to their rarity, represent a diagnostic and therapeutic challenge. This clinical case depicts an unusual metastasis pathway for an unusual neoplasm.

## INTRODUCTION

Appendicular lesions are divided into neoplastic and non-neoplastic.^1^ The former encompasses epithelial and neuroendocrine tumors, and the latter corresponds to mucoceles and inflammatory diseases. Epithelial neoplasms include polyps, mucinous adenocarcinoma of the appendix (MACA), non-mucinous adenocarcinoma of the appendix (NMACA), or colonic type, and non-invasive mucinous neoplasms of the appendix, low-grade appendiceal mucinous neoplasm (LAMN), and high-grade appendiceal mucinous neoplasm (HAMN).^2^ Neuroendocrine neoplasms include neuroendocrine tumors and mixed tumors with features of adenocarcinoma.

Appendicular neoplasms are rare, with an incidence of 0.12 per million people per year.^3^ Among carcinomas, mucinous adenocarcinomas are the most common, followed by non-mucinous and neuroendocrine carcinomas.^4^

The classification of appendix adenocarcinomas into mucinous type is given by extracellular mucin in more than 50% of the lesion in a microscopic section.^1^

The median age at diagnosis of MACA is 59 years, with a slightly higher incidence in women (55%). At diagnosis, one-third of these tumors are staged as T3, half as T4 and M1, and the majority (80%) are N0.^5^ MACA is less prone to lymph node metastases than NMACAs,^5^ and well-differentiated ones are more likely to have peritoneal spread than distant metastases.^6^ To the best of our knowledge, no case of inguinal ganglion metastasis of mucinous adenocarcinoma of the appendix has been described by searching in PubMed with the MeSH terms “appendix”, “adenocarcinoma mucinous”, “lymphatic metastasis” and “groin”.

## CASE REPORT

**Figure 1:**
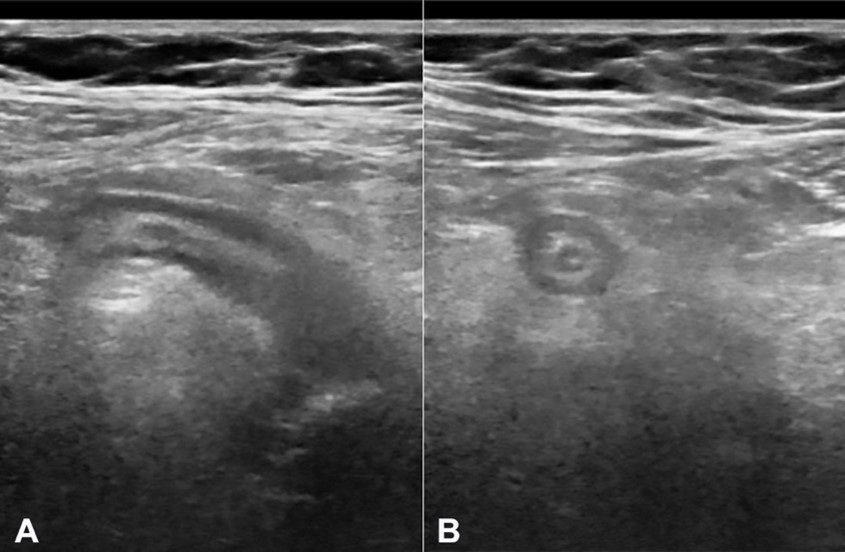
Abdominal ultrasound. Distended ileocecal appendix (13mm in diameter), with hypoechoic lumen, parietal thickening, and diffuse hyperreflectivity of the surrounding fat (**A** and **B**).

A 71-year-old woman with a history of total hysterectomy and bilateral salpingo-oophorectomy by adenomyosis with benign final pathology, 20 years ago, came to the Emergency Department for abdominal pain in the lower quadrants, of sudden onset, associated with anorexia and nausea over the last 36 hours. On the physical examination, she had hemodynamic stability, apyrexia, and abdominal right lower quadrant pain without signs of peritoneal irritation. Analytically, she had increased inflammatory parameters, and ultrasound revealed uncomplicated acute appendicitis (Figure 1).

**Figure 2:**
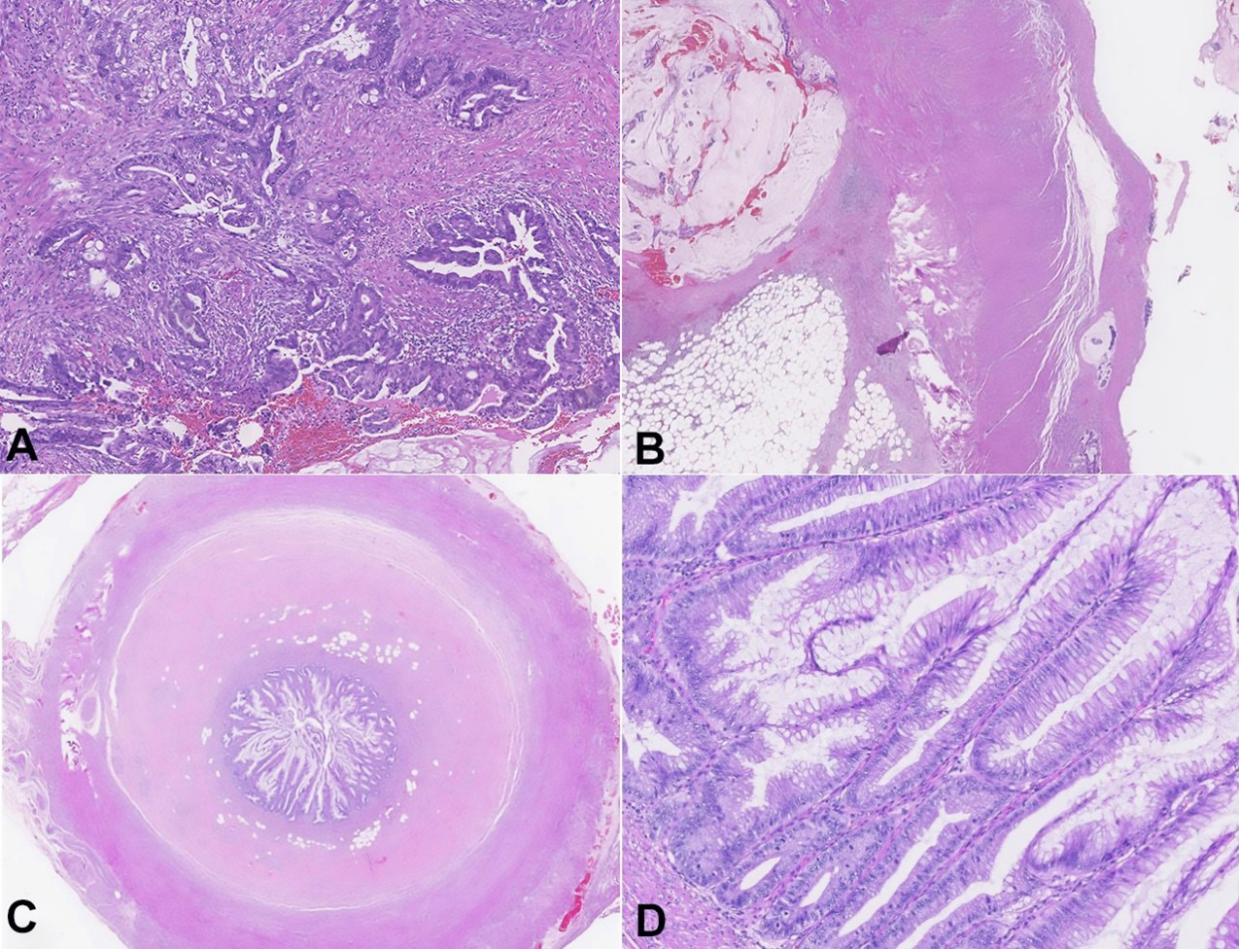
Photomicrographs of the ileocecal appendix. Area of invasive adenocarcinoma, with desmoplastic stroma and focal presence of extracellular mucus (inferior right corner) - H&E stain 10x (**A**). Appendicular wall, with neoplastic glandular structures in the subserosa (superior left corner) with a lake of mucus accompanied by rare neoplastic cell clusters (inferior left corner) - H&E stain lower magnification (**B**). Cross-section of the appendicular surgical top - H&E stain (**C**). Detail of the mucosa of the appendicular surgical top, where low-grade dysplasia lesions are observed - H&E stain 20x (**D**).

The patient underwent an uneventful laparoscopic appendectomy. Pathological examination revealed well-differentiated invasive mucinous adenocarcinoma of the appendicular middle third, 2 cm in length, with subserosa invasion, without appendicular wall rupture, with overlapping evidence of acute appendicitis and appendicular mucosa of the proximal top with low-grade dysplasia lesions (Figure 2).

Further staging by computerized tomography (CT) failed to identify metastatic disease. The carcinoembryonic antigen (CEA) and carbohydrate antigen 19-9 (CA 19-9) were normal. No other lesions were identified in the lower digestive endoscopy (LDE); thus, the patient was staged as pT3cN0M0 and underwent laparoscopic radical right hemicolectomy. Histological examination revealed lymph node substage (12 lymph nodes) without signs of malignancy, and adjuvant chemotherapy (QT) with capecitabine was proposed.

**Figure 3:**
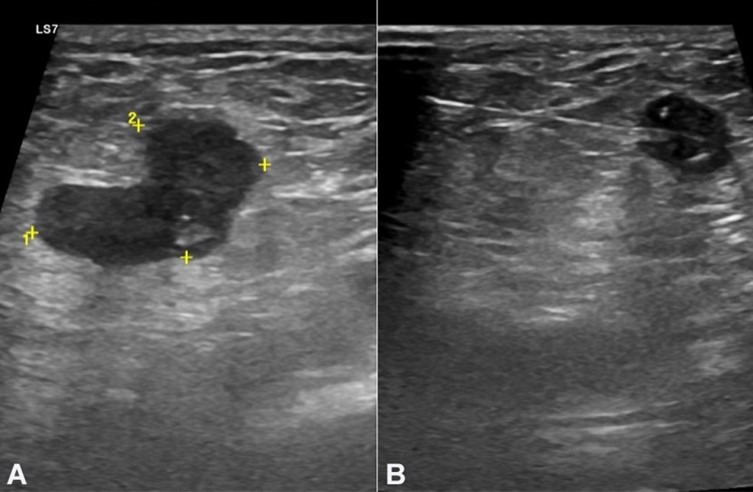
Inguinal ultrasound. Suspicious right inguinal adenomegaly measuring 20 x 11 mm (**A**) and the adenomegaly during the tru-cut biopsy (**B**).

**Figure 4:**
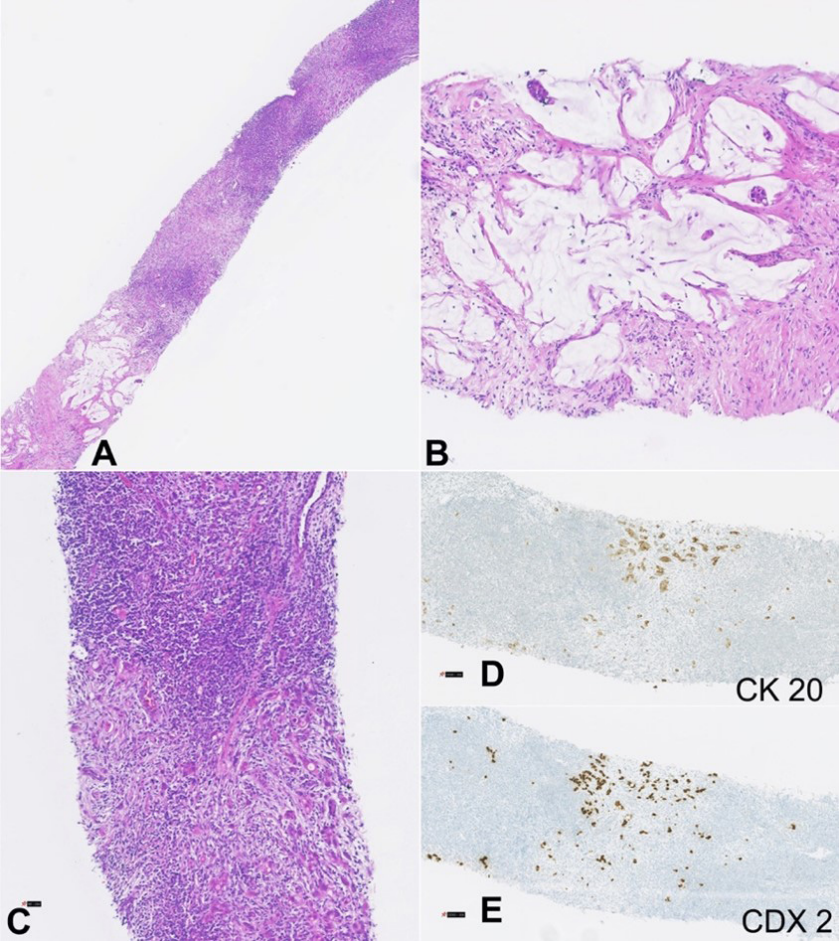
Photomicrographs of the lymph node biopsy. Fragment of a lymph node partially involved by adenocarcinoma metastasis, with growth areas in the form of small glands accompanied by stromal desmoplasia and others represented by extracellular mucus lakes accompanied by rare neoplastic glands - H&E stain lower magnification (**A**). Small neoplastic glands within a pool of extracellular mucus - H&E stain 10x (**B**). Area of lymph node tissue with multifocal metastatic involvement, due to adenocarcinoma, represented by multiple small neoplastic glands accompanied by mild desmoplastic stromal reaction - H&E stain 20x (**C**). Isolated neoplastic cells and several small neoplastic glands were highlighted by immunohistochemical staining for cytokeratin 20 (**D**) and CDX 2 (**E**) – 20x magnification.

In the third year of follow-up, the patient presented a right inguinal swelling. The ultrasound depicted two suspicious lymph nodes (Figure 3). The tru‐cut biopsy revealed adenocarcinoma, with areas of extracellular mucin and an immunophenotypic profile compatible with primary colorectal neoplasia (Figure 4). At that time, tumor markers were elevated, the LDE did not identify endoluminal lesions, and CT did not show the involvement of others lymph nodes groups. The patient was re-staged to stage IV and proposed for palliative chemotherapy with FOLFIRI and bevacizumab. Five months later, due to abdominal complaints, the patient underwent an abdominal CT that identified liver metastasis and findings suggestive of peritoneal carcinomatosis. The patient died 3 months after by digestive bleeding and suspected gastrointestinal perforation due to bevacizumab toxicity and disease progression.

## DISCUSSION

As in the present case, the adenocarcinoma of the appendix is ​​usually masked by the picture of acute appendicitis.^7^

Diagnosis and staging overlap with the other colic neoplasms. Tumor markers CEA and CA 19-9 are elevated in most advanced mucinous tumors,^8^ CT and ultrasound do not distinguish between neoplastic and non-neoplastic appendicular mucinous lesions, MRI is superior in detecting extraluminal mucin and peritoneal disease,^9^ and positron emission tomography is not recommended because of the high incidence of false negatives.^10^ LDE is indicated for screening for cecal involvement and synchronous colic lesions, present in up to 42% of these patients.^11^ Echo-endoscopy can be helpful in the differential diagnosis of appendicular lesions, but percutaneous biopsy is contraindicated due to the risk of peritoneal dissemination.

The treatment of appendix adenocarcinoma depends largely on the diagnostic timing (not suspected, suspected, or confirmed) or the surgical regimen (urgent or elective). In an emergency context, the gold standard is therapeutic appendectomy for patients with a clinical picture of acute appendicitis. Given the suspicion of intraoperative neoplasia, the extent of surgery should be dictated by the ability to obtain macroscopic free margins, ranging from resection of the caecum to ileocolectomy, or right hemicolectomy. If peritoneal disease is suspected, cytoreductive surgery is not indicated *ad initium*. In elective surgery, diagnostic appendectomy is indicated for mucinous lesions whenever the preoperative neoplastic diagnosis is not available. In situations with preoperative confirmation, right hemicolectomy should be performed in localized disease, coupled with cytoreductive surgery with hyperthermic intraperitoneal chemotherapy (HIPEC) in metastatic disease. In cases of contained rupture, right hemicolectomy should be the norm, and in free rupture, this should be associated with peritoneal lavage and peritoneal biopsies of suspect lesions.

Well-differentiated MACA is associated with a low risk of lymph node metastasis (6% T1, 0% T2, 7% T3, 22% T4), and there seems to be no significant difference in the survival of patients undergoing appendectomy or radical right hemicolectomy.^5^ Therefore, patients undergoing R0 appendectomy (margin-negative resection) for well-differentiated tumors, T1 or T2, can be kept under surveillance, leaving the right hemicolectomy for the remaining cases, as in the clinical case described (T3 and proximal margin with low-grade dysplasia).^12^

The role of radiotherapy and adjuvant chemotherapy is not established. Extrapolating from the efficacy of adjuvant chemotherapy with fluorouracil (FU) and oxaliplatin for N+ colon adenocarcinoma cases, adjuvant chemotherapy appears to be a valid option in adenocarcinomas of the appendix with lymph node invasion.^12^ In the present case, the lymph node substage dictated the need for adjuvant chemotherapy. According to the literature, 5-year survival of MACA patients varies between 37% and 69%^5^.

In the presented case, it is hard to say if the inguinal lymph node metastasis were an extension of the peritoneal involvement or if they already existed at the time of the appendectomy. Initially, the patient had only inguinal lymph node involvement, and the findings suggestive of peritoneal carcinomatosis, which was not confirmed, was related to a TC performed 5 months after the diagnosis of inguinal involvement.

## CONCLUSION

Appendicular neoplasms, due to their rarity, represent a diagnostic and therapeutic challenge. This clinical case depicts an unusual metastasis pathway for an unusual neoplasm.
